# Antigen Design for Successful Isolation of Highly Challenging Therapeutic Anti-GPCR Antibodies

**DOI:** 10.3390/ijms21218240

**Published:** 2020-11-03

**Authors:** Man-Seok Ju, Sang Taek Jung

**Affiliations:** 1 Department of Biomedical Sciences, Korea University College of Medicine, Seoul 02841, Korea; seok0801@korea.ac.kr; 2 Institute of Human Genetics, Korea University College of Medicine, Seoul 02841, Korea; 3 Department of Biomedical Sciences, Graduate School, Korea University, Seoul 02841, Korea; 4 BK21 Graduate Program, Department of Biomedical Sciences, Korea University College of Medicine, Seoul 02841, Korea; 5 Biomedical Research Center, Korea University Anam Hospital, Seoul 02841, Korea

**Keywords:** G protein-coupled receptor, membrane protein, antigen, therapeutic antibody

## Abstract

G-protein-coupled receptors (GPCR) transmit extracellular signals into cells to regulate a variety of cellular functions and are closely related to the homeostasis of the human body and the progression of various types of diseases. Great attention has been paid to GPCRs as excellent drug targets, and there are many commercially available small-molecule chemical drugs against GPCRs. Despite this, the development of therapeutic anti-GPCR antibodies has been delayed and is challenging due to the difficulty in preparing active forms of GPCR antigens, resulting from their low cellular expression and complex structures. Here, we focus on anti-GPCR antibodies that have been approved or are subject to clinical trials and present various technologies to prepare active GPCR antigens that enable the isolation of therapeutic antibodies to proceed toward clinical validation.

## 1. Introduction

**Figure 1:**
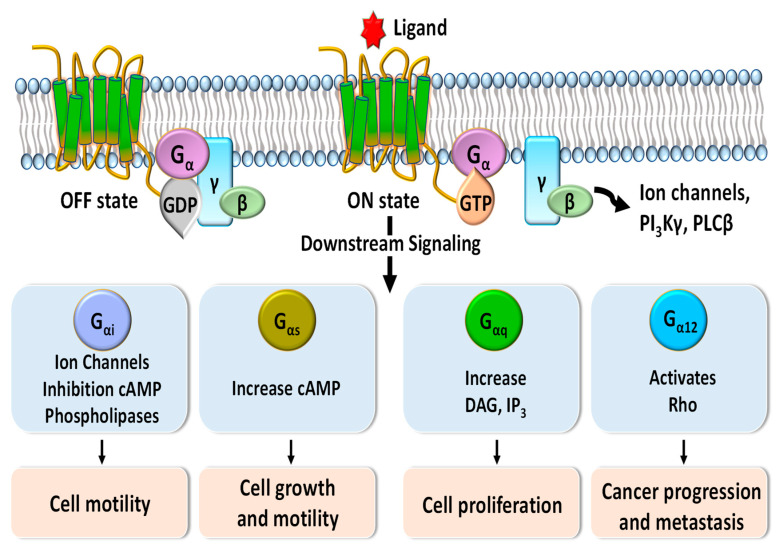
Schematic diagram of G-protein-coupled receptor (GPCR) signaling pathways mediated by G_α_ protein subunits. Downstream signaling triggered by binding of G proteins changes the concentrations of phospholipase C-beta (PLCβ), phosphoinositide 3-kinases-gamma (PI3Kγ), diacylglycerol (DAG), inositol trisphosphate (IP3), and cyclic adenosine monophosphate (cAMP) and regulates various cellular functions such as cell motility, cell growth, cell proliferation, and cancer progression and metastasis.

G-protein-coupled receptors (GPCRs), which make up the largest superfamily of human membrane proteins, play pivotal roles in mediating intracellular signaling and inducing cell proliferation, cell growth, and cell motility through the association and subsequent dissociation of G-proteins in response to external stimuli (Figure 1) [1,2]. Many clinical studies have revealed that abnormal functions of GPCRs are highly related to a variety of human diseases and affect the patient survival rate [3,4,5]. Therefore, GPCRs are crucial drug targets to treat patients with various diseases, and their targeting drugs represent more than 30 percent of all US Food and Drug Administration (FDA)-approved drugs [6,7,8]. Annual sales of these drugs have increased to about USD 180 billion in 2018 [9].

**Figure 2:**
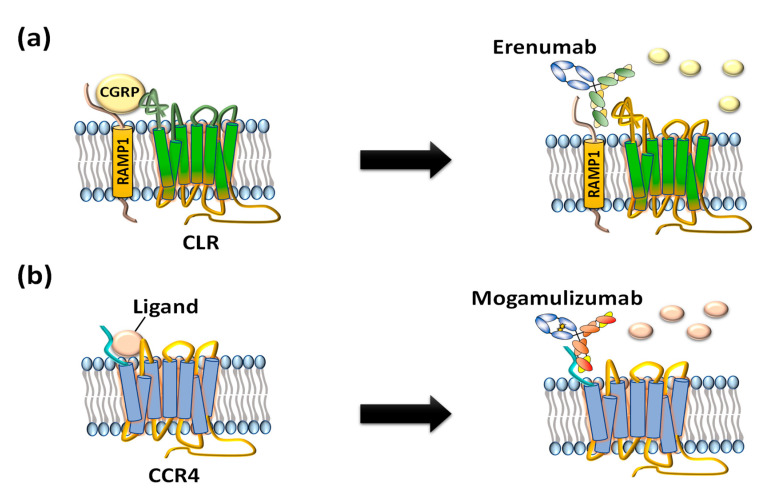
US FDA-approved anti-GPCR antibodies erenumab and mogamulizumab. (**a**) Erenumab is an antagonistic monoclonal antibody against calcitonin gene-related peptide receptor (CGRPR) consisting of calcitonin receptor-like receptor (CLR) and receptor activity-modifying protein 1 (RAMP1) for treatment of chronic migraine. (**b**) Mogamulizumab is an antibody against chemokine receptor 4 (CCR4) for treatment of T-cell leukemia by inactivating the GPCR and clearance of target cells by enhanced antibody-dependent cell-mediated cytotoxicity (ADCC).

Compared to small-molecule chemical drugs and small peptides, therapeutic antibodies have many advantages in terms of higher target specificity, fewer side effects, and superior serum circulating half-life [10]. However, despite clinical and marketing successes of monoclonal antibody products to treat numerous diseases, only two anti-GPCR therapeutic antibody drugs, Amgen’s erenumab (trade name: Aimovig), targeting calcitonin gene-related receptor (CGRPR) to treat migraine (Figure 2a) [11] and Kyowa Kirin’s mogamulizumab (trade name: Poteligeo), targeting chemokine receptor 4 (CCR4) to treat refractory mycosis fungoides and Sézary syndrome (Figure 2b), have been approved to date [12].

Generally, amenable techniques to isolate therapeutic human antibodies include (1) humanization of candidate antibodies followed by the selection of hybridoma cells derived from immunized animals; (2) screening of the human naïve antibody library displayed on the surface of bacteriophages, bacteria, or yeast, which take advantage of a physical linkage between genotype and phenotype; and (3) hybridoma selection after immunizing an antigen into humanized transgenic animals, referred to as XenoMouse^TM^, which contains the genes for variable regions of the heavy (VH) and light (VL) chains of the human antibody repertoire [13].

Regardless of the antibody isolation technique, preparing pure GPCR antigens with the native conformation of the human in vivo condition is essential for successful isolation of therapeutic functional human anti-GPCR antibodies. In particular, GPCRs containing seven transmembrane α-helices are usually expressed at very low levels in heterologous expression systems; therefore, it is very hard to purify the antigen with a native conformation as a soluble form. Furthermore, the limited surface area of the extracellular region of GPCRs in the whole GPCR structure makes it very difficult to prepare the GPCR antigen as a target for therapeutic anti-GPCR antibodies. Even though antigen preparation is one of the most difficult steps in development of therapeutic anti-GPCR antibodies, two anti-GPCR antibodies have overcome the challenges and have been recently approved. In addition, dozens of anti-GPCR antibodies are under clinical development or are waiting for clinical evaluations of therapeutic efficacy and toxicity. In this review, we focus on therapeutic anti-GPCR antibodies that have been recently approved or those that are subject to clinical trials (Table 1) and how the various types of GPCR antigens are prepared to isolate the highly challenging therapeutic anti-GPCR antibodies that have entered the clinical development phase.

## 2. GPCR Extracellular Region Fusion Proteins as GPCR Antigens

**Figure 3:**
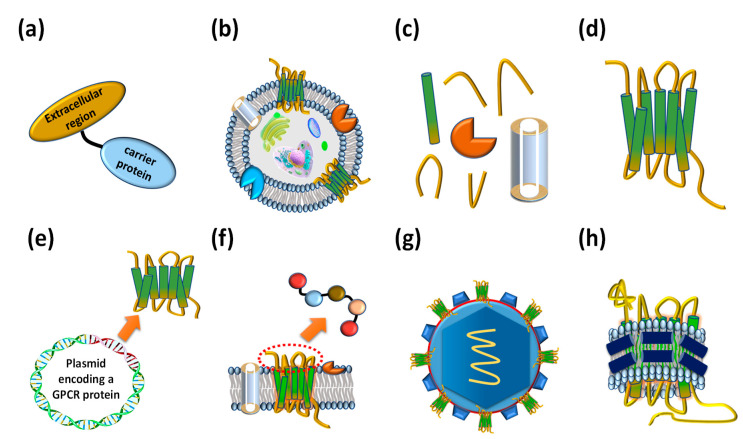
Various types of antigens that have been employed to isolate anti-GPCR antibodies. (**a**) Extracellular region fused proteins. (**b**) Whole cells expressing GPCRs on their cellular membrane. (**c**) Membrane factions expressing GPCRs. (**d**) Purified whole GPCRs. (**e**) DNA molecules encoding GPCRs. (**f**) Extracellular region peptides. (**g**) Virus-like particles (VLPs) displaying GPCRs on the surface. (**h**) Reconstituted GPCR–lipid–belt protein (GLB) complexes.

Interaction between the extracellular region of GPCRs and their ligands triggers conformational changes in the intracellular region of the protein, resulting in the association of G-proteins and the transmission of extracellular signals into cells [26]. GPCRs consist of seven transmembrane α-helical bundles, four extracellular regions: N-terminal, extracellular loop 1 (ECL1), extracellular loop 2 (ECL2), and extracellular loop 3 (ECL3), and four intracellular regions: intracellular loop 1 (ICL1), intracellular loop 2 (ICL2), intracellular loop 3 (ICL3), and C-terminal. The unique three-dimensional structure of each GPCR determines its ligand specificity to elicit its characteristic cellular responses. To isolate antibodies that recognize the extracellular region of a GPCR, a simple strategy is to chemically conjugate or genetically fuse a designed peptide comprising the part of the GPCR extracellular region with a carrier protein (Figure 3a). The prepared antigen can be used to immunize animals or screen antibodies from a human naïve antibody library [27]. Although the extracellular region peptide prepared with a carrier protein is unable to perfectly mimic the extracellular peptide conformation of a native GPCR, some human GPCR extracellular region polypeptides containing post-translational modifications such as glycosylation can be expressed in mammalian cells [28]. A mimic GPCR extracellular loop was fused with a carrier protein and employed to isolate erenumab, targeting calcitonin gene-related peptide receptor (CGRPR) [29], mogamulizumab, targeting chemokine receptor 4 (CCR4) [30], and vantictumab, targeting Frizzled-7 (FZD7) [31].

Erenumab is an antibody developed to treat patients with chronic migraine. It regulates the function of calcitonin receptor-like receptor (CLR), interacting with the receptor activity-modifying protein (RAMP) family. The single transmembrane domain has selectivity for three types of ligands: calcitonin gene-related peptide (CGRP), adrenomedullin 1, and adrenomedullin 2 [32]. The calcitonin gene-related peptide receptor (CGRPR), comprising CLR and RAMP1, is mainly distributed in the peripheral and central nervous systems.

**Figure 4:**
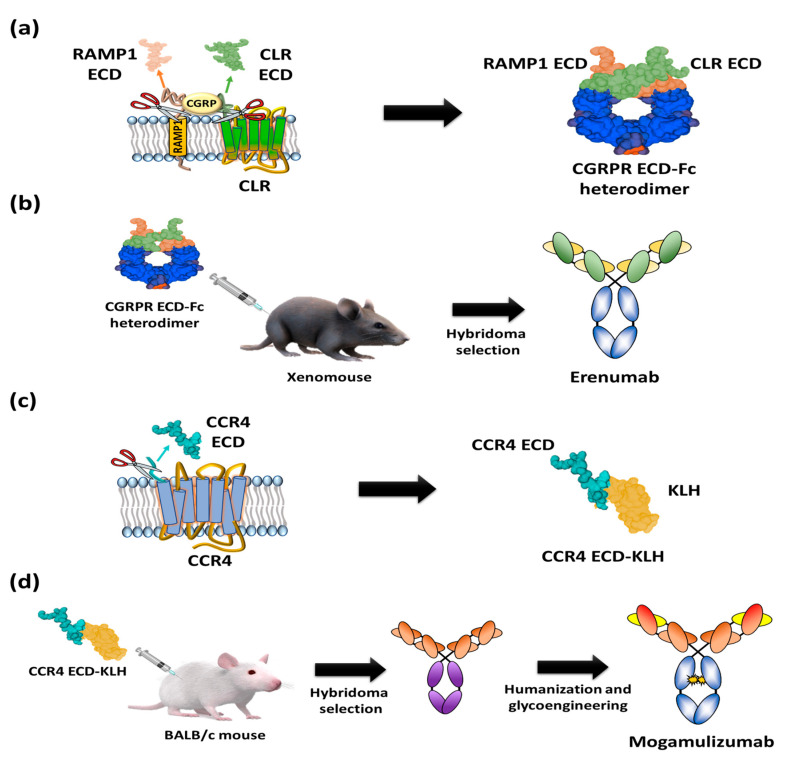
Schematic diagrams of antigen preparation and overall procedure for discovery of US FDA-approved anti-GPCR antibody. (**a**) A heterodimer CGRPR Fc protein prepared by Fc fusion constructs of N-terminal extracellular regions of CLR and ectodomain of RAMP1. (**b**) Immunization of heterodimeric CGRPR Fc proteins into XenoMouse hybridoma selection to isolate erenumab. (**c**) The N-terminal region of CCR4 (28 amino acids: N2-C29) fused with keyhole limpet hemocyanin (KLH). (**d**) Injection of the N-terminal region of CCR4-KLH into BALB/c mice and hybridoma selection, humanization, and defucosylation of N-inked glycans of Fc to generate mogamulizumab.

Moreover, it is closely related to migraine through vasodilation following G_αs_ protein release and the activation of adenylyl cyclase [33,34]. Based on the finding that the CGRP binding site spans the extracellular regions of CLR and RAMP1 [35], a heterodimeric Fc (fragment crystallizable) fusion protein consisting of an ectodomain of RAMP and an N-terminal extracellular region of CLR was designed as an antigen to isolate CGRPR antagonistic antibodies (Figure 4a). In addition, the prepared heterodimeric Fc fusion was immunized into XenoMouse to generate erenumab, followed by hybridoma screening [36,37] (Figure 4b). Erenumab showed a high binding affinity to CGRPR (K_D_ = 56 pM) and excellent inhibition of cAMP production (IC_50_ = 2.3 nM) [38]. Additionally, structural analysis of the CGRPR-CGRP complex confirmed that erenumab directly blocks the conformation of CGRP into CGRPR [39]. In the phase III clinical trial, patients treated with 70 mg and 140 mg of erenumab once a month for at least 6 months showed 43.3% and 50% reductions of number of days of migraine [40], respectively, and these efficacy results enabled the antibody to be the first anti-GPCR antibody approved by the US FDA in May 2018 and by the European Medicines Agency (EMA) in July 2018.

Mogamulizumab is a humanized monoclonal antibody targeting chemokine receptor 4 (CCR4) with a glyco-engineered Fc region to enhance the antibody-dependent cellular cytotoxicity (ADCC) for the clearance of adult t-cell leukemia (ATL). The protein CCR4 is overexpressed on the surface of FOXP3+ regulatory T (Treg) cells of ATL patients and is involved in the evasion of immune surveillance against tumors [41,42]. To isolate anti-CCR4 antibodies, extracellular partial N-terminal peptide (28 amino acids: N2-C29) was fused to a carrier protein, keyhole limpet hemocyanin (KLH) (Figure 4c), and injected into mice for the screening of antigen-specific antibodies (Figure 4d) [43]. After humanization of the candidate antibodies, glycol engineering was performed to defucosylate the N-linked glycan of Fc to enhance FcγIIIa binding and Natural Killer (NK) cell-mediated ADCC activity [44,45,46,47]. The resulting mogamulizumab (KW-0761) exhibited significant efficacy in 50% of ATL patients treated in clinical trials, leading to successful commercialization in 2012 in Japan and in 2019 in the US for the treatment of mycosis fungoides (MF) and Sézary syndrome (SS) [12,48].

Uncontrolled cell signaling in the Wnt/β-catenin pathway affects various types of tumors [49]. Vantictumab (anti-FZD7), a Wnt/β-catenin pathway-blocking antibody, was isolated by screening the human naïve Fab antibody library displayed on bacteriophages using the Fc-fused extracellular N-terminal domain of the GPCR as an antigen. The resulting antibody (vantictumab) could inhibit Wnt pathway signaling through specific binding to five kinds of Frizzled receptors: FZD1, FZD2, FZD5, FZD7, and FZD8 [31]. In human phase 1 clinical examination, vantictumab significantly inhibited the growth of pancreatic, colon, and breast cancer cells in combination with other chemotherapeutic agents [50].

## 3. GPCR-Expressing Cells or Membrane Fractions as GPCR Antigens

The use of GPCR-expressing cells (Figure 3b) or their membrane fractions as antigens containing integral and associated proteins (Figure 3c) has been limited due to difficulty in overexpressing a target GPCR on the cell surface due to the presence of numerous other membrane components. In addition, a very advanced handling technique is necessary to apply the fragile whole cells as antigens for repeated rounds of antibody screening. Alternatively, the membrane fraction from cells displaying a target GPCR has been used as a type of antigen. The biggest advantage of using GPCR-expressing cells or membrane fractions is their native conformation that allows for the isolation of a desired anti-GPCR antibody capable of recognizing the native structure of GPCR compared to using other GPCR mimetic antigens.

Representative examples of therapeutic anti-GPCR antibodies that have been isolated using animal immunization with GPCR-overexpressing cells or their membranes fraction as antigens are glutazumab, targeting glucagon-like peptide-1 receptor (GLP1R) [51,52], volagidemab, targeting glucagon receptor (GCGR) [53], plozalizumab, targeting chemokine receptor type 2 (CCR2) [54], leronlimab (Pro 140), targeting C-C chemokine receptor type 5 (CCR5) [55], ulocuplumab (BMS-936564), targeting C-X-C chemokine receptor type 4 (CXCR4) [56], and avdoralimab (IPH5401), targeting C5a receptor (C5aR) [57].

Glutazumab is an agonist antibody targeting GLP1R for the treatment of type 2 diabetes resulting from an abnormal cellular response to insulin. It was developed by hybridoma selection from mice that were immunized with GLP1R-expressing mammalian cells, humanization, and genetic fusion of the GLP1 (29 amino acids: H7-G35), a ligand of GLP1R, at the N-terminus of the variable light chain of IgG [51,58]. Glutazumab was efficacious in suppressing the interaction between GLP-1 and GLP1R and showed significant efficacy in suppressing glucagon secretion in a human phase II clinical trial in Australia and New Zealand [23].

Leronlimab (Pro 140) is a humanized monoclonal antibody targeting CCR5, which is expressed on the surface of T lymphocytes and is essential for the fusion of HIV with immune cells. Anti-CCR5 antibodies were isolated by immunizing mice with CCR5-expressing mammalian cells, and humanization of the resulting antibodies enabled the development of leronlimab (Pro 140) [55]. The antibody inhibits HIV infection pathways by selectively binding to the N-terminus and extracellular loop 2 of CCR5, and a human phase III clinical trial is ongoing [19,59].

## 4. Purified Whole GPCR Proteins as GPCR Antigens

For preparative production of whole GPCR proteins (Figure 3d) that mimic the native GPCR structure, several strategies, including the optimization of expression conditions, detergents to extract the complicated membrane proteins, and purification, have been attempted. Despite reports of the successful production of functional GPCRs in mammalian, insect, and Escherichia coli host cells, their preparation techniques are highly variable depending on the type of GPCR. In addition, the same ligand binding affinity and specificity for purified GPCRs as those on cellular membranes are not guaranteed because the structures extracted from cellular membranes are likely to be different from those of native GPCRs. Nevertheless, the use of purified whole GPCR proteins as antigens enables the exclusion of a number of unrelated components on the cellular membrane, which may improve the isolation of GPCR target-specific antibodies. For the efficient production of functional native-like GPCRs, significant efforts have aimed to resolve issues including the (i) low expression level of GPCR on the cell membrane surface, (ii) low solubility and stability of expressed GPCRs, and (iii) complicated reconstitution steps to maintain the active conformation of GPCRs.

To improve the expression level of endothelin receptor type A (ET_A_) in *E. coli*, Lee et al. fused the N-terminus of a GPCR with the P9 peptide derived from an envelope protein of Pseudomonas phage Φ6 (Phi6). The P9 peptide fusion significantly increased ET_A_ expression in *E. coli*, and the purified ET_A_ showed binding affinity to both its native ligand (ET-1 peptide) and G_α_ protein [60]. Corin et al. added non-ionic detergents in a commercial cell-free translation system to successfully express and purify 13 GPCRs. Human vomeronasal receptor 1 (hVN1R1), prepared through its modified cell-free translation system, showed similar ligand binding affinity compared to the hVN1R1 counterpart produced in HEK293 cells [61]. For enhanced expression of GPCRs, Sarkar et al. optimized the conditions to enhance outer membrane permeability for the access of additional small ligands to the GPCRs expressed on the *E. coli* inner membrane. Using the screening of an error-prone PCR library for rat neurotensin receptor-1 (NTR1), they successfully isolated NTR1 variants exhibiting an improved fluorescence signal upon binding to fluorescent dye-conjugated ligands in the flow cytometric analysis. The resulting GPCR variants showed enhanced stability and were successfully purified in *E. coli*, yeast, and mammalian cells [62]. A. James Link et al. fused Green Fluorescent Protein (GFP) to the C-terminus of GPCRs and monitored the effect of co-expression of a panel of selected *E. coli* proteins on GPCR expression. They found that co-expression of membrane-anchored AAA+ protease FtsH could significantly improve the expression levels of full-length cannabinoid receptors (CB_1_, CB_2_) and bradykinin receptor 2 (BR2) in *E. coli* [63]. Vukoti et al. examined the effect of detergents on the solubility of recombinant purified cannabinoid receptor 2 (CB_2_) using the *E. coli* expression system and optimized conditions for the reconstitution of CB_2_ from mixed n-dodecyl-ß-D-maltopyranoside (DDM), 3-[(3-cholamidopropyl) dimethylammonio]-1-propanesulfonate (CHAPS), and cholesteryl hemisuccinate (CHS) micelles. The reconstituted CB2 exhibited an equivalent binding affinity to ligands (CP-55,940 or SR-144,528) and G_αi1_ compared to the CB2 expressed in Chinese Hamster Ovary (CHO) cells [64].

## 5. Other Types of Prepared GPCR Antigens

In addition to the previously mentioned types of antigens, other types of prepared GPCR antigens such as DNA (Figure 3e), peptide (Figure 3f), virus-like particles (VLP) (Figure 3g), and the GPCR-lipid-belt protein (GLB) complex (Figure 3h) have been harnessed to isolate therapeutic anti-GPCR antibodies. The biggest advantage of using GPCR-coding DNAs as antigens is to bypass production of complex GPCR membrane proteins or GPCR-overexpressing cells. When the DNA encoding the GPCR antigen is cloned into a vector optimized for GPCR protein expression and injected into an animal host, it produces heterologous GPCR protein antigen and GPCR overexpressing cells. The resulting GPCR antigen enables to isolate GPCR antigen specific antibodies through the immune response of the animal host [65,66] However, injected DNAs are highly labile to decomposition by the immune responses of animals. In addition, it is hard to isolate a desired target-specific anti-GPCR antibody in the case of low antigenic GPCR expression. Peptide antigens can be effectively used when the structures of the antigen proteins are complex or protein production is very difficult. And GPCR-specific antibodies can be discovered using peptides consisting of the sequence of the extracellular loop of the GPCR [67,68]. However, it is not easy to prepare the peptide antigens that mimic the native GPCR extracellular loop structure, and the short-length peptide antigen may be labile to be degraded in the host when it is immunized into an animal. VLPs are divided into two main types, non-enveloped VLPs and enveloped VLPs, depending on the presence of a lipid bilayer on the VLP surface displaying antigens [69]. Enveloped VLP containing a lipid bilayer structure can display complex membrane proteins on the cell surface and has been used to isolate anti-GPCR antibodies [70,71]. However, it is possible that the conformation and topology of GPCRs displayed on the surface of VLP differ from those of natural GPCR expressed on cellular membrane.

Similarly, the GPCR-lipid-belt protein (GLB) complex prepared by reconstituting solubilized GPCR proteins, lipids, and belt proteins has been used as an antigen to isolate various anti-GPCR antibodies [72,73]. Namacizumab, a therapeutic antibody for treatment of non-alcoholic fatty liver disease (NAFLD), was isolated using the prepared GLB complex reconstituted with belt protein, lipid, and cannabinoid 1 receptor (CNR1) [74]. CNR1 is a main receptor for anandamide and 2-arachidonoyl glycerol and is involved in energy metabolism [75,76]. Previous research results indicate that overexpression and dysfunction of CNR1 cause obesity and hepatic steatosis (fatty liver disease) in a diet-induced obesity mouse model [77]. The anti-CNR1 antibody was isolated by injecting reconstituted CNR1 GLB complex particles into mice [74,78], and a phase I human clinical trial of its humanized antibody was conducted [25].

## 6. Conclusions

In contrast to small-molecule drugs, monoclonal antibodies have many advantages, including high target specificity, much reduced side effects, prolonged serum half-life, and the ability to harness immune effector functions mediated by a variety of leukocytes. Due to these unique characteristics possessed by antibodies, they are the fastest growing sector in drug development. GPCRs are crucial for regulating the cell growth, motility, proliferation, progression, and metastasis of cancer. Although many small-molecule drugs targeting GPCRs are commercially available, only two anti-GPCR therapeutic antibody drugs have been approved by the US FDA as of 16 September 2020. One of the main reasons for the slow speed of development of therapeutic antibodies against the attractive drug target is the difficulty in preparing functional (native or native-like) GPCR antigens. It is evident that GPCRs are highly challenging antigens for which to isolate therapeutic antibodies because of their complex structure. However, as noted above, various strategies to prepare homogeneous and more native-like GPCR antigens have been developed. In keeping pace with the development of GPCR antigen preparation methods, various cutting-edge antibody isolation platforms have emerged. Our research group also discovered novel anti-GPCR antibodies for cancer treatment through the innovative methods described in this manuscript, and the results will be reported soon. In combination with efficient GPCR antigen preparation methods and advanced antibody screening techniques, many advances have been achieved and will soon be able to exploit a variety of anti-GPCR therapeutic antibodies with new mechanisms of action, which may be used for the treatment of a variety of diseases related to GPCR signaling.

## Figures and Tables

**Table 1:** Anti-GPCR antibodies approved by the US FDA or subject to clinical trial.

Antigen Type	Antibody Screening Technology	Drug Name	Target	Indication	Status of Clinical Trial	Reference
RAMP1-Fc, CGRPR-Fc	Tg mouse immunization and hybridoma screening	Aimovig^TM^ (Erenumab)	CGRP receptor	Migraine	Approved	[14]
CCR4-KLH	Mouse immunization and hybridoma screening	Poteligeo^®^ (Mogamulizumab)	CCR4	Refractory mycosis fungoides and Sézary disease	Approved	[15]
FZD7-Fc	Synthetic human antibody phage library screening	Vantictumab (OMP-18R5)	FZD7	Non-small cell lung cancer, pancreatic cancer, metastatic breast cancer	Phase I	[16,17,18]
CCR5 expressing cells	Mouse immunization and hybridoma screening	Leronlimab (Pro 140)	CCR5	HIV infection	Phase III	[19]
CCR2 expressing cells	Mouse immunization and hybridoma screening	Plozalizumab (MLN-1202)	CCR2	Relapsing-remitting multiple sclerosis	Phase II	[20]
CXCR4 expressing cells	Tg mouse immunization and phage library screening	Ulocuplumab (BMS-936564)	CXCR4	Acute myeloid leukemia	Phase I/II	[21]
C5Aa1 expressing cells	Tg mouse immunization and hybridoma screening	Avdoralimab (IPH5401)	C5aR1	Non-small cell lung cancer, liver cancer	Phase I	[22]
Membrane fraction of GLP1R expressing cells	Tg mouse immunization and hybridoma screening	Glutazumab (GMA102)	GLP1R	Type 2 diabetes	Phase II	[23]
Membrane fraction of GCGR expressing cells	Tg mouse immunization and hybridoma screening	Volagidemab (REMD-477)	GCGR	Diabetes mellitus	Phase II	[24]
Reconstituted CNR1 GLB complex	Mouse immunization and hybridoma/phage library screening	Namacizumab (RYI-018)	CNR1	Non-alcoholic fatty liver disease	Phase I	[25]
